# College Libraries

**Published:** 1888-12

**Authors:** 


### EDITORIAL.

### College Libraries.

A Literary or Scientific School or College would hardly be regarded as fully or even well equipped without a library.

Every Literary College of even an ordinary reputation and status has its general library, and it is a point always aimed at, to make it as complete as possible.

No Scientific School would attain the desired success, nor command general respect without its special library.

Why Professional Colleges should be exceptional in this respect, it is difficult to understand.

All Theological Schools have their special libraries, and would not be sustained without.

Law Schools have their libraries as a necessary attachment.

A few Medical Colleges have good libraries, some have a few books without much regard to selection. And many have not even an apology for a library, the students relying wholly upon their own text books.

Our Dental Colleges are also very deficient in regard to libraries; but few having even a beginning for a library.

Access to the literature of the profession is a very desirable and profitable thing, especially for senior students, it is an important aid in the prosecution of their more advanced studies, and not only this, but they become familiar with the literature of the profession, and become interested in, and appreciate the right use of it, and in the future will be lead to regard a good library as one of the important adjuncts of a truly professional man.

It is to be hoped that in the future Dental Colleges will give more attention to this matter than in the past.

